# Open versus robot-assisted retroperitoneal tumors resection involving inferior vena cava, abdominal aorta, and renal hilum: a comparative study

**DOI:** 10.1007/s00464-024-10848-1

**Published:** 2024-04-24

**Authors:** Manan Sulaiman, Khan Akhtar Ali, Yang Chunguang, Rubina Hashim, Yang Luan, Ze Zhong Xiong, Hui Huang, Zhihua Wang

**Affiliations:** 1 Department of Urology, Tongji Hospital, Tongji Medical College, Huazhong University of Science and Technology, Wuhan, 430030 China; 2 Department of Trauma, Shaheed Mohtarma Benazir Bhutto Institute of Trauma, Karachi, 74000 Pakistan; 3 Department of Orthopedic Surgery, Tongji Hospital, Tongji Medical College, Huazhong University of Science and Technology, Wuhan, 430030 China

**Keywords:** Robotic surgery, Retroperitoneal tumors, Benign tumors, Malignant tumors, Complications

## Abstract

**Introduction:**

Surgery is currently the only effective treatment for retroperitoneal tumors that do not involve any specific organ. The use of robots for removing both benign and malignant retroperitoneal tumors is considered safe and feasible. However, there is insufficient evidence to determine whether robotic retroperitoneal tumor resection (RMBRs) is superior to open retroperitoneal malignant resection (OMBRs). This study compares the short-term outcomes of robotic excision of benign and malignant retroperitoneal tumors with open excision of the same-sized tumors.

**Methods:**

The study compared demographics and outcomes of patients who underwent robotic resection (*n* = 54) vs open resection (*n* = 54) of retroperitoneal tumors between March 2018 and December 2022. A 1:1 matching analysis was conducted to ensure a fair comparison.

**Results:**

The study found that RBMRs resulted in reduced operative time (OT), estimated blood loss (EBM), and postoperative hospital stay (PSH) when compared to OBMRs. Additionally, RBMRs reduced EBL, PHS, and OT for patients with malignant tumor involvement in major vessels. No significant differences were found in tumor size, blood transfusion rate, and morbidity rate between the RBMRs and OBMRs groups.

**Conclusion:**

When comparing RMBRs to OMBRs, it was observed that RMBR was associated with lower (EBL), shorter postoperative hospital stays (PHS), and reduced operative time (OT) in a specific group of patients with both benign and malignant tumors.

**Supplementary Information:**

The online version contains supplementary material available at 10.1007/s00464-024-10848-1.

Retroperitoneal tumors (RT) are mesenchymal tumors that can be benign or malignant. They develop in the retroperitoneal region and have no confirmed organ connection [1, 2]. Retroperitoneal tumors can be either benign or malignant, and they differ in various ways, such as their depth of attachment to significant vessels and operating space. The retroperitoneal great vessels are poorly understood, but they can potentially complicate surgical procedures and negatively impact treatment outcomes [3–7]. The retroperitoneum is a rare site for tumor growth, which can arise from various soft tissues and affect retroperitoneal organs. Retroperitoneal tumors can be benign or malignant, with malignant tumors further classified into those originating from epithelial tissues and those arising from non-epithelial tissues. Liposarcoma, leiomyosarcoma, and undifferentiated pleomorphic sarcoma are the most common subtypes. Certain types of sarcomas are more common in children, while diseases characterized by lymph node enlargement primarily affect adults. It is important to acquire a reliable diagnosis before considering surgery because the therapeutic techniques, including medical and surgical treatment, of malignant and benign tumors and the type and scope of surgery might differ significantly among the various kinds of tumors. A large number of benign and malignant tumors are eradicated through a laparotomy, which extends the hospital stay following surgery, postoperative agony, and delays healing [8–11]. A reliable diagnosis is crucial before surgery, as treatment options for malignant and benign tumors vary significantly. Robotic techniques are increasingly used in retroperitoneal surgeries due to limited space and advanced surgical equipment. These techniques have a shorter learning curve and result in better postoperative recovery, less blood loss, and shorter operation times compared to open surgeries [12] Robotic resection for tumors has limited published reports, mostly case studies. Despite advancements in robotic techniques, research on this topic is still limited [13–18]. Our study aims to compare the outcomes of robotic and open excision of retroperitoneal tumors. We hypothesize that robotic-assisted techniques will result in less operative time, bleeding, and postoperative complications, as well as minimal recurrence of tumors.

## Patients and methods

We conducted a cross-sectional study to analyze the outcomes of surgical treatments performed between March 2018 and December 2022. During this period, we performed robotic resection on 104 patients and open resection on 68 patients. Before the surgery, experienced surgeons evaluated each patient to determine the best surgical option (robotic or open). All patients who were registered for the study were considered suitable candidates for both robotic and open surgery. They were given detailed information about the benefits and drawbacks of each surgical approach before making their final selection. There was no difference in tumor size between both groups. In this work, we applied propensity score matching to mitigate selection bias. Following the matching process, there were no obvious differences in demographic characteristics between the robotic and open groups. Patients provided their informed consent before the surgery and gave written consent for this study afterward. To compare the outcomes of robotic and open surgeries, we conducted a study with 1:1 propensity score analysis and telephonic interview were also performed. We matched 54 patients who had robotic surgery with 54 patients who had open surgery based on age, sex, BMI, ASA score, tumor size, and the procedure itself. The hospital ethical committee approved and registered this study.

## Preoperative evaluation

Retroperitoneal tumors are detected through imaging and histological analysis. Ultrasound is the first method used, but its subjectivity is a drawback. Contrast-enhanced CT is preferred for assessing and staging primary retroperitoneal masses. Radiological indicators help establish a differential diagnosis, including the beak sign, phantom organ sign, embedded organ sign, and prominent feeding artery sign. CT angiography or 3D reconstruction is used for preoperative evaluation and surgical planning.

## Inclusion and exclusion

To be eligible for inclusion, the patient must be between 18 and 82 years old, have a resect able retroperitoneal tumor (malignant or benign), and not have any medical conditions that would make anesthesia or surgery unsafe. Exclusion criteria include tumors bigger than 10 cm, no evidence of tumor involvement in the retroperitoneal organs, and severe cardiopulmonary or hepatorenal insufficiency. Surgery may still be considered if the tumor is adhering to major blood vessels.

### Perioperative data

Data from preoperative and pathology records, along with baseline demographics, were collected. The following parameters were analyzed: operation time, estimated blood loss, blood transfusion, conversion to open surgery, postoperative complications, and PHS. Mortality and readmission rates within 1 year were also investigated. Pathology records were examined for tumor information.

### Surgical technique and follow-up

**Fig. 1:**
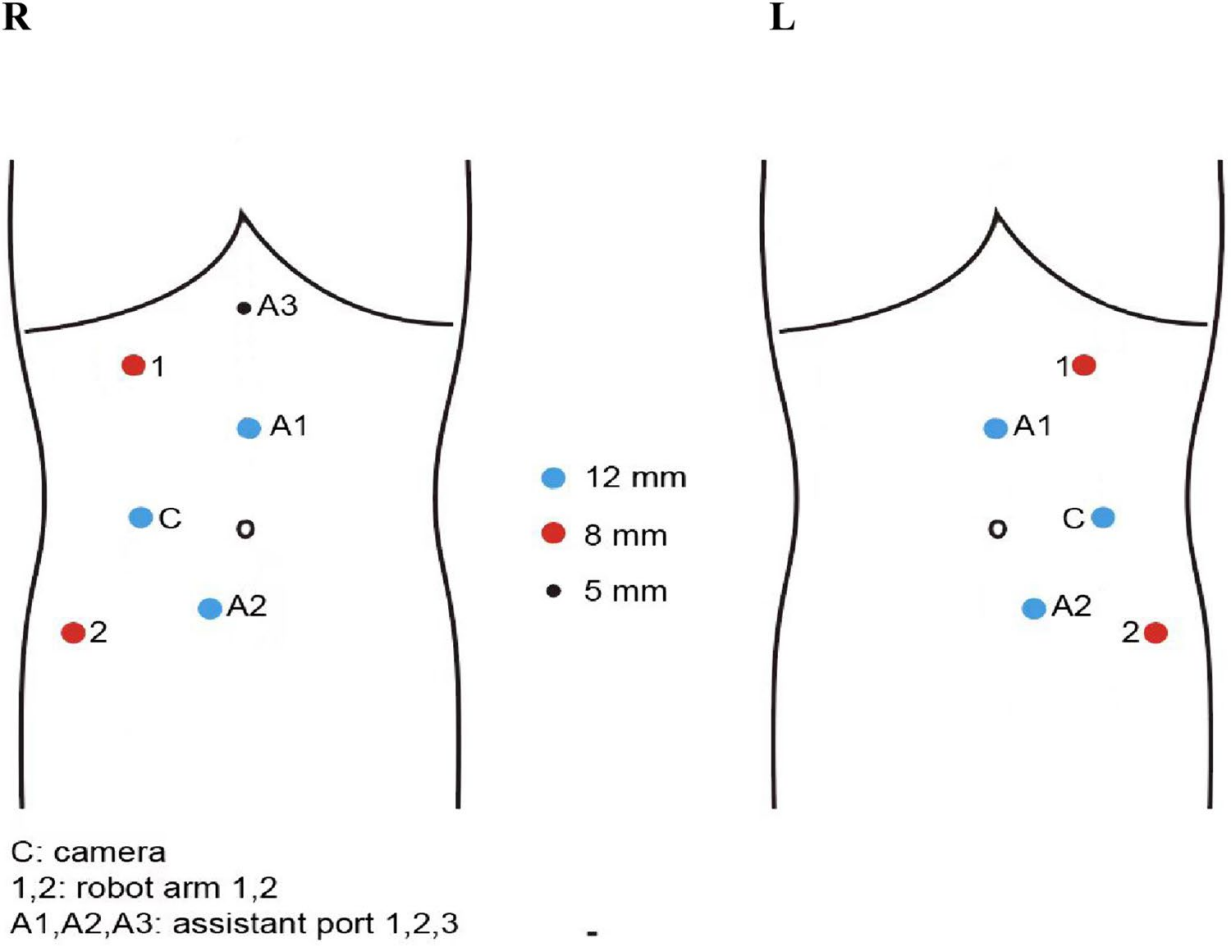
Port placement for robotic tumor excision. Trocar placement for the surgery. A six-trocar technique was applied for the surgery. One 12 mm trocar near the umbilicus was the designated camera port (point C). Camera port, robot arm port 1 (near the liver margin) and 2 (above the anterior superior iliac spine) formed approximate 120 angular degree. Assistant port A1and A2 were 8cm from the camera port. A 5 mm trocar under the liver margin near the midclavicular line was used as the assistant port for liver retraction (point A3)

RBMR surgeries were performed by an experienced team with over 1000 robotic procedures using the Da Vinci Xi Surgical System. The same team performed both robotic and open surgeries. For robotic resection the patient was positioned in the left lateral decubitus position with the right side elevated approximately 70 degrees during the surgery. They were under general anesthesia and continuously monitored with central venous and arterial pressure monitoring Fig. 1.

To ensure an open approach, the patient is carefully placed in the right lateral Jackknife position at a 90-degree angle. The support for this is provided by both a beanbag and an axillary role. To ensure protection, the left arm is equipped with ample padding as well as an airplane-type retractor. It is important to secure the left arm such that the scapula rolls anteriorly. The incision will be made overlying the 11th or 12th intercostal space and extended toward the umbilicus. The position shifted in accordance with the tumor’s left or right location. Follow-up data was collected through outpatient departments and phone interviews. In the first year, patients underwent clinical examinations, blood tests, and imaging every 3 months.

### Statistical analysis

Continuous data were reported as either mean SD or median based on their distributions. Categorical data were compared using the chi-square test. To reduce the impact of possible confounders and selection bias, propensity score analysis was used to match covariates such as age, sex, body mass index (BMI), ASA score, tumour size, and tumour involvement to major blood vessels between the two groups. A 1:1 nearest neighbour matching method was used to select individuals for each group. All statistical tests were performed using SPSS v22.0 (SPSS Inc., Chicago, IL, USA). A *p*-value of less than 0.05 was considered statistically significant.

## Results

During the study period, 172 patients (104 RBMR and 68 OBMRs) had retroperitoneal benign and malignant tumors excision. Patients in the OBMRs group had a bigger tumors size and more blood transfusions than those in the RBMRs group before matching. In this propensity score matching trial, 54 patients RBMRs and 54 patients received OBMRs. After matching, there were not any significant differences in demographic variables between the two groups.

### Pathological and postoperative outcome

Upon final histological inspection, it was found that the tumors in both groups were a mix of benign and malignant. Table 1 shows that both groups had similar pathology. Table 2 displays the perioperative results for both groups. The robotic group had significantly reduced surgical time (*p* = 0.004), lower estimated blood loss (*p* = 0.008), and shorter postoperative hospital stay (*p* = 0.001) compared to the open group. There was a significant postoperative complication, with 3 patients from each group experiencing lymphatic leakage and receiving treatment in accordance with hospital guidelines. Both groups had similar rates of blood transfusion (*p* = 0.7504). All patients underwent effective robotic removal of both benign and malignant tumors located in the retroperitoneal region, with no need to switch to laparotomy. The mortality rate in both groups was 1.8%. There were no patients who needed to undergo another operation or were readmitted to the hospital within 1 year.

**Table 1:** Comparison of robotic group and open group: patients characteristics

Characteristics	Before propensity score matching			After propensity score matching		
	Robotic (*N* = 104)	Open (*N* = 68)	*p* value	Robotic (*N* = 54)	Open (*N* = 54)	*p* value
Age	50.00 ± 14.12	49.88 ± 13.72	0.9570	49.67 ± 12.82	51.02 ± 13.00	0.5876
Male (%)	52 (50)	34 (50)	0.4611	26 (50)	26 (50)	
Female	52 (50)	34 (50)		26 (50)	26 (50)	
BMI	23.54 ± 2.853	23.28 ± 2.766	0.5577	23.09 ± 2.895	23.08 ± 2.663	0.9906
ASA. score classification						
2 (%)	89 (79)	52 (73.52)	0.1287	40 (70.37)	40(72.22)	0.999
3 (%)	15(13)	16 (23.52)		14 (25.92)	14 (25.92)	
Anatomical tumor laterally						
Right (%)	47 (45)	30 (44)	0.9172	21 (38)	22 (40)	0.9780
Left (%)	44 (42)	28 (41)		24 (43)	25 (40)	
Bilateral (%)	13 (13)	10 (15)		9 (19)	7 (20)	
Pre operative tumor size	6.664 ± 1.909	7.394 ± 2.007	0.0147	7.026 ± 1.891	6.393 ± 1.463	0.0542
Blood transfusion, *N* (%)	9 (8.6)	14 (20.5)	0.0245	6 (11.1)	5 (9.2)	0.7504
Vessel’s attachment, *N* (%)						
Right renal arteries and veins	22 (21.1)	12 (17.6)		13 (24.0)	11 (20.3)	
Left renal arteries and veins	26 (25)	15 (22.0)		15 (27.7)	14 (25.9)	
Inferior vena cava, right renal veins and artery	19 (18.3)	12 (17.6)		8 (14.8)	10 (18.5)	
Inferior vena cava, left renal veins and artery	18 (17.3)	17 (25)		10 (18.5)	11 (20.3)	
Abdomen aorta, Inferior vena cava, right renal veins and artery	12 (11.5)	7 (10.2)		4 (7.4)	4 (7.4)	
Abdomen aorta, Inferior vena cava, left renal veins and artery	5 (4.8)	5 (7.3)		4 (7.4)	4 (7.4)	
Splenic veins Left renal artery and veins	2 (4.8)	0 (0)		0 ()	0 ()	
Tumor pathology						
Schwannoma (%)	13 (12.5)	9 (13.2)		8 (14.8)	7 (12.9)	
Lymphoma (%)	21 (20.2)	10 (14.7)		10 (18.5)	10 (18.5)	
Castleman disease (%)	2 (1.9)	1 (1.5)		1 (1.8)	2 (3.7)	
epidermoid cyst (%)	3 (2.8)	1 (1.5)		1 (1.8)	1 (1.8)	
Ganglioneuroma (%)	8 (7.7)	4 (5.9)		3 (5.5)	4 (7.4)	
Paraganglioma (%)	11 (10.5)	9 (13.2)		7 (12.9)	6 (11.1)	
Neuroblastoma (%)	3 (2.9)	3 (4.4)		3 (5.5)	2 (3.7)	
Teratoma (%)	7 (6.7)	7 (10.3)		3 (5.5)	7 (12.9)	
Hemangioma (%)	6 (5.7)	4 (5.9)		3 (5.5)	1 (1.8)	
Lymphangioma (%)	6 (5.7)	2 (2.9)		3 (5.5)	2 (3.7)	
Leiomyosarcoma (%)	2 (1.9)	2 (2.9)		2 (3.7)	1 (1.8)	
Fibrous malignant tumor (%)	6 (5.7)	4 (5.9)		2 (3.7)	4 (7.4)	
Liposarcoma (%)	4 (3.8)	3 (4.4)		1 (1.8)	3 (5.5)	
spindle cell malignant tumor (%)	4 (3.8)	3 (4.4)		2 (3.7)	2 (3.7)	
Myxoid (%)	8 (7.7)	6 (8.8)		5 (9.2)	2 (3.7)	

Values represent mean ± SD, or *N* (%). Boldface represents statistically value

*N/A* not applicable

**Table 2:** Comparison of robotic group and open group: postoperative outcomes

Characteristic	Robotic (*N* = 54)	Open (*N* = 54)	*p* value
Operative time	137.9 ± 46.93	171.0 ± 46.59	0.0004
Estimated blood loss ML	192.0 ± 185.4	316.4 ± 190.7	0.0008
Blood transfusion, *N* (%)	6 (11.1)	5 (9.2)	0.7504
Postoperative hospital stays	4.056 ± 0.9793	5.556 ± 1.355	0.0001
Lymphatic leak, *N* (%)	3 (5.5)	3 (5.5)	
Injury to adjacent organs, *N* (%)	0 (0)	0(0)	N/A
Conversion to open surgery *N* (%)	0 (0)	0 (0)	N/A
Morbidity *N* (%)	8 (14.81)	10 (18.51)	0.6056
One year readmission (%)	0 (0)	0 (0)	N/A
One year reoperation (%)	0 (0)	0 (0)	N/A
One year Mortality (%)	1 (1.8)	1 (1.8)	

Values represent mean ± SD, or *N* (%). Boldface represents statistically value

*N/A* not applicable

### Clinical outcomes of malignant tumors involvement the major vessels

Table 3 summarizes the characteristics of patients with malignant tumors involving major vessels before undergoing surgery. The data shows that both the Robotic and Open surgery groups had similar age, sex, BMI, and tumor size. They also had comparable rates of blood transfusion, illness, and mortality. The rates of positive surgical margin, local recurrence, adjuvant chemotherapy, radiotherapy, and target therapy were all similar between both groups. However, there were significant differences between the two groups in terms of estimated blood loss (EBL) (*p* = 0.0221), postoperative hospital stays (PHS) (*p* = 0.0001), and operation time (OT) (*p* = 0.0023).

**Table 3:** Comparison of robotic versus open resection for tumors involving major vessels, Including demographic and perioperative outcomes

Characteristics	After propensity score matching malignant tumors		
	Robotic (*N* = 12)	Open (*N* = 12)	*p* value
Age	50.17 ± 43.70	43.83 ± 14.73	0.2873
Male (%)	6 (50)	6 (50)	0.9999
Female	6 (50)	6 (50)	
BMI	22.71 ± 2.669	23.20 ± 2.855	0.6646
ASA. score classification			0.6733
2 (%)	7 (58)	8 (67)	
3 (%)	5 (42)	4 (33)	
4 (%)	0 (0)	0 (0)	
Pre operative tumor size	8.142 ± 1.780	6.442 ± 1.242	0.2734
Estimated blood loss ML	191.7 ± 193.1	442.5 ± 241.7	0.0221
Blood transfusion, *N* (%)	3 (25)	3 (25)	
Operative time MIN	148 .2 ± 20.98	171 .7 ± 49.14	0.0023
Postoperative hospital stays	4 .000 ± 0.6030	6.083 ± 1.240	0.0001
Morbidity *N* (%)	3 (25)	2 (16.7)	0.6152
Positive surgical margins rate, *N* (%)	7 (50)	5 (41.7)	
ADJUVANT CHEMOTHERAPY, *N* (%)	5 (41.7)	4 (33.3)	
Three cycles (%)	1 (8.3)	2 (16.7)	
Four cycles (%)	3 (25)	2 (16.7)	
Five cycles (%)	1(8.3)	0 (0)	
ADJUVANT RADIOTHERAPY, *N* (%)	1 (8.3)	1 (8.3)	
ADJUVAT TARGRTED THERAPY, *N* (%)	2 (16)	0 (0)	
Local recurrence	3 (25)	4 (33)	
One year readmission (%)	0 (0)	0 (0)	N/A
One year reoperation (%)	0 (0)	0 (0)	N/A
One year mortality (%)	1 (8.3)	1 (8.3)	

Values represent mean ± SD, or *N* (%). statistically value

*N/A* not applicable

## Discussion

The findings of our study Robotic surgery group compared to the open surgery group. The robotic group showed significantly reduced PHS, intraoperative blood loss, and operating time. Increased accuracy and delicacy during manipulation may lessen surgical harm. All RBMRs in this study were performed exclusively utilizing the fully robotic method, without any need for conversion to open surgery. All patients who were registered for the study were considered suitable candidates for both robotic and open surgery. They were given detailed information about the benefits and drawbacks of each surgical approach before making their final selection. There was no difference in tumor size between both groups. In this work, we applied propensity score matching to mitigate selection bias. Following the matching process, there were no obvious differences in demographic characteristics between the robotic and open groups. Robotic surgery has gained popularity due to its ability to enhance manipulation capabilities, provide better visibility, and enable rapid bleeding management. It also decreases injury to the gastrointestinal system and expedites the recovery of gastrointestinal function as compared to open surgery [19–24]. Additionally, the results after surgery are consistently getting better and it is widely accepted. Robotic surgery is rapidly becoming the preferred method in urology experiencing substantial growth in industrialized countries. That they have exceeded the outcomes of traditional open surgery for most treatments [25]. Robotic resection techniques are safe and feasible for removing retroperitoneal tumors, including large tumors or those attached to major blood arteries, with no significant negative impact on perioperative outcomes [26]. Due to the greater probability of malignant retroperitoneal tumors invading major vasculature, we further categorized the patients based on the tumor type. Our findings indicate that robotic surgery considerably reduced estimated blood loss (EBL) and postoperative hospital stay (PHS) in these patients as compared to open surgery patients. The enhanced dexterity and visualization provided by the robotic technology can be credited for its positive impact on tumor dissection, tissue mobilization, and vascular control. Retrospective and randomized controlled trials provide ample evidence indicating that use of minimally invasive techniques for treating early-stage lung cancer, bladder cancer, cervical cancer offers numerous clinical benefits in terms of perioperative outcomes compared to open surgery [27–29]. Robotic retroperitoneal surgery resulted in significantly less blood loss than open retroperitoneal surgery for both benign and malignant cases. Traditional open or laparoscopic surgical procedures, such as partial nephrectomy, radical nephrectomy, retroperitoneal lymph node dissection, nephroureterectomy, and adrenalectomy, are now frequently performed with robot assistance. Robot-assisted retroperitoneal oncological surgery is continually evolving, with a special focus on preserving oncological and functional results while reducing surgical invasiveness [16, 30]. Additionally, there was no significant difference observed in the rates of postoperative morbidity between RMBR and OMBR. This finding implies that robotic resection of malignant tumors measuring no larger than 10 cm seems to be technically safe and efficacious. Robotic surgery resulted in faster recovery than open surgery in both our study and the study conducted by Giulianotti PC et al. Patients who underwent robotic surgery had early bowel function recovery, minimal analgesic needs, and did not require blood transfusions. All patients resumed a normal diet by day two, and most experienced flatulence and bowel movement within the first day. Robotic surgery is a safe option for benign and malignant tumor resection [31]. In this trial, 172 patients were studied, out of which 104 underwent entirely robotic treatment without requiring open surgery. The robotic group showed a significantly shorter postoperative stay when compared to the open group in our study. Our study demonstrated statistically significant differences in operation times and shorter recovery times for robot-assisted retroperitoneal tumor removal when compared to the open retroperitoneal tumor resection group. Robotic surgery offers improved dexterity, intuitive instrument handling, tremor reduction, motion scaling, and superior 3D visualization [32, 33]. As surgeons gain more experience in operating robotic systems, they tend to become more efficient, which results in shorter operative times. Robotic systems provide better precision, dexterity, and visualization when compared to traditional open surgery, which allows for more accurate and efficient complex procedures. However, there may be a learning curve for surgeons and their teams when adopting robotic surgery. As the experience of surgeons increases, the time taken for operations tends to decrease. Moreover, this study has shown that there were statistically significant differences in the blood transfusion rates between the two patient groups.

## Limitations

This study has several limitations, including a relatively small sample size, limited evaluation of long-term outcomes, and lack of information about the surgeons' experience level with robotic surgery. Additionally, the study did not evaluate the cost-effectiveness of robotic surgery or potential confounding factors that may impact the outcomes. Future studies should address these limitations by conducting larger-scale investigations that evaluate the long-term effects of robotic surgery on patients, assess the cost-effectiveness of the procedure, and account for potential confounding factors. Furthermore, future studies should evaluate the feasibility and safety of robotic surgery for retroperitoneal tumors in a variety of settings and patient populations. By addressing these limitations and exploring future perspectives, researchers can continue to improve the outcomes and accessibility of surgical treatments for retroperitoneal tumors.

## Conclusion

In conclusion, our findings show that RMBRs are as safe and effective as OMBRs in patients with retroperitoneal benign and malignant tumors. With the benefits of the robotic approach, RMBRs may offer significant improvements over OMBRs, reducing OT, EBL and PHS in patients even for tumors involving major vessels.

### Supplementary Information

Below is the link to the electronic supplementary material.

Supplementary file1 (mp4 2239 KB)

## Data Availability

Researchers who make a valid request to the corresponding author will be given access to the data.
